# Percentage of culture confirmation and melting curve analysis reveals false-positive *Campylobacter* detection in a molecular syndromic panel

**DOI:** 10.1128/jcm.00028-25

**Published:** 2025-07-14

**Authors:** Annie Sanchez, Michael Rauch, Sherry Buechner, Brittney Jung-Hynes, Eric Beck, Allen Bateman

**Affiliations:** 1 Laboratory Leadership Service, Centers for Disease Control and Prevention 1242, Atlanta, Georgia, USA; 2 Communicable Disease Division, Wisconsin State Laboratory of Hygiene, Madison, Wisconsin, USA; 3 University/Hospital and Clinics, UW Health Clinical Laboratories, Madison, Wisconsin, USA; 4 Aurora West Allis Medical Center, ACL Laboratories, West Allis, Wisconsin, USA; Endeavor Health, Evanston, Illinois, USA

**Keywords:** CIDT, *Campylobacter*, *Salmonella*, STEC, BioFire, FilmArray, gastrointestinal infection

## Abstract

Surveillance of enteric organisms can identify outbreaks and support control measures. The BIOFIRE FilmArray Gastrointestinal Panel (FilmArray GI) is the most common culture-independent diagnostic test (CIDT) used in Wisconsin to diagnose enteric infections. State clinical laboratories have raised concerns about inaccurate *Campylobacter* detection with the FilmArray GI. The Wisconsin State Laboratory of Hygiene (WSLH) evaluated the percentage of positivity and culture confirmations stratified by CIDT for *Campylobacter*, *Salmonella*, and STEC from 2018 to 2024. We further analyzed the transport time of *Campylobacter* specimens and the melt curves of specimens that tested positive by FilmArray GI. *Campylobacter*, *Salmonella*, and STEC specimens tested on the FilmArray GI had increases in percentage of positivity compared to those tested on other CIDTs. *Salmonella* and STEC specimens positive by FilmArray GI, compared to other CIDTs, had no significant culture confirmation differences. However, *Campylobacter* specimens positive by FilmArray GI had significantly lower culture confirmations than positive specimens from other CIDTs (62% vs. 78%; *P* < 0.05). *Campylobacter c*ulture confirmations were lower for FilmArray GI specimens, compared to other CIDTs, regardless of how many days the specimen spent in transport in 2023. Additionally, we found atypical melt curves among specimens positive by FilmArray GI with cultures that did not grow *Campylobacter*. Taken together, the higher percentage of positivity, the lower percentage of culture confirmation, and the atypical melt curves suggest that the FilmArray GI might yield false-positive *Campylobacter* results. These findings emphasize the importance of public health surveillance to identify potential issues with commercially available diagnostic tests.

**IMPORTANCE:**

Enteric pathogens cause ~9.4 million illnesses annually in the United States. Clinical laboratories rely on culture-independent diagnostic testing platforms (CIDTs) for rapid and accurate diagnosis of enteric pathogens. *Campylobacter*, *Salmonella*, and Shiga-toxin-producing *Escherichia coli* (STEC) are three of the most identified enteric bacterial infections in Wisconsin. The BioFire FilmArray Gastrointestinal panel (FilmArray GI) is currently the most common CIDT used by Wisconsin clinical laboratories to diagnose *Campylobacter*, *Salmonella*, and STEC infections. However, the FilmArray GI has had notable issues and recalls in the past. Here, we used public health surveillance data to assess platform performance for these organisms. We analyzed percent positivity and culture confirmations based on testing platforms. Through our analysis, we identified potential false-positive *Campylobacter* results from the FilmArray GI, which were associated with atypical melt curve profiles on the BioFire platform. Public health surveillance can help identify potential issues with diagnostic platforms.

## INTRODUCTION

*Campylobacter* is a bacterium that affects the intestinal tract and occasionally the bloodstream of infected persons. *Campylobacter* infections cause approximately 1.5 million illnesses and 25,000 hospitalizations in the United States annually (1, 2). *Campylobacter* is among the most common reportable bacterial enteric pathogens in Wisconsin (3). Most *Campylobacter* infections occur by eating undercooked poultry products containing the bacteria, but can be found in other sources (e.g., contaminated water) (1, 3).

In Wisconsin, campylobacteriosis is a reportable condition with approximately 1,500 *Campylobacter* diagnoses every year (4). Clinical laboratories across the state routinely perform diagnostic tests for stool specimens from patients with clinical presentations consistent with enteric infections. *Campylobacter* can be diagnosed by stool culture or using culture-independent diagnostic platforms (CIDTs). During the past 10 years, CIDT use among clinical laboratories increased nationally (5–9). The BIOFIRE FilmArray (bioMérieux Inc., Marcy-l'Étoile, France) Gastrointestinal panel (FilmArray GI) is a commonly used CIDT platform. The FilmArray GI is the dominant platform used to diagnose enteric pathogens, including by 523 of 696 (75%) laboratories that performed the College of American Pathology (CAP) GI Panel, 5 Challenge (GIP5) proficiency testing in 2024. Although this is a commonly used platform, the U.S. Federal Drug Administration issued recalls of the FilmArray GI for false-positive *Cryptosporidium* and *Campylobacter* in 2019, and more recently, *Norovirus* in 2024 (10, 11). Atypical melt curve profiles were associated with false-positive *Norovirus* and *Cryptosporidium* results using FilmArray GI (12, 13).

A large clinical laboratory in Wisconsin contacted the Wisconsin State Laboratory of Hygiene (WSLH) because their percentage of culture-confirmed *Campylobacter* CIDT-positive specimens was lower when they switched their molecular diagnostic platform to the FilmArray GI in 2023. A second Wisconsin clinical laboratory also noted a substantial decrease in *Campylobacter* culture confirmations when they switched their CIDT platform to the FilmArray GI later that year. These findings led WSLH to use a multi-modal approach to investigate *Campylobacter* percent positivity, culture confirmation rates, specimen time in transport, and FilmArray GI melt curves.

## MATERIALS AND METHODS

This study was reviewed by CDC and was conducted consistent with federal law and CDC policy (*45 C.F.R. part 46, 21 C.F.R. part 56; 42 U.S.C. Sect. 241(d); 5 U.S.C. Sect. 552 a; 44 U.S.C. Sect. 3501 et seq).

### Clinical laboratory data to determine percent positivity

Clinical laboratory surveillance data from patients with diarrhea symptoms seeking standard-of-care diagnosis from January 1, 2018, to June 30, 2024, were used to investigate organism percent positivity. WSLH requests that clinical laboratories in Wisconsin submit weekly molecular diagnostic surveillance data through the Wisconsin Clinical Laboratory Network (WCLN). This state-wide surveillance request is open to all clinical laboratories performing molecular diagnostic testing. Requested data include the number of specimens tested, number of positive specimens, and CIDT used (14). We evaluated CIDT percent positivity of the most common reportable bacterial enteric pathogens in Wisconsin: *Campylobacter*, *Salmonella*, and Shiga-toxin producing *Escherichia coli* (STEC). Results from the FilmArray GI platform were compared to results from other CIDTs in aggregate. The CIDTs were BD Max Enteric Bacteria Panel (Becton, Dickinson and Company, Sparks, MD), Verigene Enteric Pathogens Nucleic Acid Test, and xTAG Gastrointestinal Pathogen Panel (Luminex Corporation, Austin, TX). Surveillance data without CIDT information, or data with the FilmArray GI listed together with another CIDT, were not included in the percent positivity analyses (36% of total clinical laboratory data submitted was excluded). Percent positivity was calculated by dividing the number of positive tests by the total number of specimens tested by FilmArray GI vs. other CIDTs. Quarterly and yearly percent positivity rates were calculated.

### Enteric pathogen culture

Clinical laboratories submit positive CIDT specimens to WSLH for culture and further characterization. WSLH requested that primary stool specimens be transported in enteric transport medium (Para-Pak, Cary Blair, or equivalent) at room temperature, according to manufacturer’s instructions, with refrigeration preferred if in transit >24 hours. Stool specimens were streaked onto appropriate selective media and observed for growth. To culture *Campylobacter*, stool was plated onto Cefoperazone, Vancomycin, Amphotericin B Agar (CVA) and incubated in *Campylobacter* jars with altered atmosphere (10% hydrogen, 10% CO2, and nitrogen balance). Stool was also inoculated onto blood agar plates (BAP) by placing specimens on a 0.65 µm MCE membrane filter disc applied directly on the agar and incubated at 35–37°C for 15 minutes. The filter disc was removed from plates before incubation in *Campylobacter* jars with the altered atmosphere described above. CVA plates were incubated at 42°C, whereas BAP was incubated at 35–37°C. Plates were examined for growth after 72 hours of incubation. If no suspicious growth was observed, plates were re-incubated for another 72 hours at respective temperatures. Suspicious growth was screened with the oxidase test. Oxidase-positive organisms were identified using Bruker MALDI-TOF mass spectrometry (MALDI Biotyper® RTC library, 2021 version).

To culture *Salmonella* and STEC, stool was inoculated onto appropriate enrichment broth (Selenite broth for *Salmonella* and Gram-negative broth for STEC) and streaked onto selective media plates (MacConkey agar (MAC), Xylose Lysine Deoxychocolate agar (XLD), Hektoen Enteric agar (HE), and Bismuth Sulfite agar (BS) (if suspected *S*. Typhi) for *Salmonella*, and Sorbitol-MacConkey agar (SMAC) and Cefixime Tellurite Sorbitol-MacConkey agar (CT-SMAC) for STEC). Enrichment broth was incubated at 37°C with shaking for 18–24 hours, then streaked onto selective media plates. Plates were incubated at 37°C for 24 hours. For *Salmonella*, if no suspicious growth was observed, plates were incubated an additional 24 hours. *Salmonella* isolates were subcultured to BAP and identified using MALDI-TOF. For STEC culture, plates were screened for the presence of possible *E. coli* O157 colonies. Any *E. coli* O157 colonies (identified through slide agglutination with *E. coli* O157 antisera) were subcultured to BAP plates. If no O157 colonies were present, two morphologically distinct beta-hemolytic colonies were subcultured to BAP plates. STEC polymerase chain reaction (PCR) was performed on subcultured isolates.

### Percent culture confirmation analysis

We analyzed culture results of specimens submitted to WSLH to assess the culture confirmation rates of CIDT-positive specimens. Data from clinical laboratories in Wisconsin that submitted > 500 specimens (top submitters) in the 5-year study period were used for this analysis. Seven clinical laboratories met the quantity criteria. We collected information on CIDT platform usage for these laboratories. To calculate the percent culture confirmation value, the number of specimens that had a culture with bacterial growth was divided by the total number of specimens tested per laboratory (FilmArray GI users vs. other CIDT). Welch’s *t-*test was used to calculate quarterly and yearly percent culture confirmations.

We also analyzed the time *Campylobacter* specimens spent in transport to assess if differences contributed to specimen stability that affected culture confirmation results. We used data from specimens submitted from January 2023 to December 2023 by top submitters to compare transport times for FilmArray GI specimens and those diagnosed using other CIDTs. Time in transport was calculated using the difference between the date collected at the clinical laboratory and the date received at WSLH. A logistic regression (generalized linear model) was used to test whether the success rates for the FilmArray GI and other CIDTs (in aggregate) are significantly different while accounting for variations across days.

### Melt curve analysis

Recent publications described atypical melt curves responsible for producing false-positive *Norovirus* and *Cryptosporidium* results on FilmArray GI (12, 13). Therefore, we analyzed FilmArray GI-produced melt curves to understand their role in false-positive *Campylobacter* results. Two clinical laboratories using the FilmArray GI panel provided melt curve results for positive *Campylobacter* specimens. Melt curve analyses were used to verify target-specific amplification. The BIOFIRE FilmArray Torch System software was used to view melting curve results. We evaluated melt curves from 65 specimens: 36 from laboratory 1 and 29 from laboratory 2. We used WSLH culture results to categorize melt curves as either culture-confirmed or culture-negative specimens. Cultured-confirmed results included *C. jejuni*, *C. helveticus* or *C. upsaliensis* (*C. helveticus*/*upsaliensis*; not differentiated by MALDI-TOF), or *C. coli* species. Melt curves from FilmArray GI-positive specimens with confirmed culture growth were compared to FilmArray GI-positive specimens that were culture-negative. Melt curve results were used to assess typical melting temperature profiles for *Campylobacter* species in the specimens that were culture-confirmed. Melt curves from specimens with cultures that did not have growth were assessed for atypical melt curves, defined as curves that differed from typical melting temperature profiles for *Campylobacter* species.

## RESULTS

### Increased percent positivity with use of FilmArray GI

**Fig 1:**
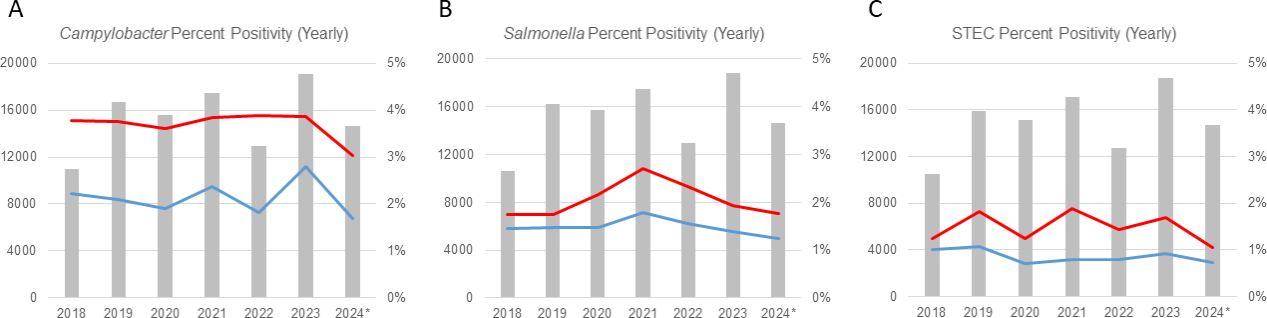
Comparison of yearly percent positivity between the FilmArray GI and other CIDTs. Percent positivity for *Campylobacter*, *Salmonella*, and STEC based on WCLN surveillance from January 2018 to June 2024. (**A–C**) The primary Y*-*axis displays the total number of specimens from clinical laboratories surveillance data analyzed (gray bars). The secondary Y*-*axis represents yearly percent positivity by FilmArray GI (red) compared to other CIDTs (blue). *Analysis done up to June 30, 2024.

We analyzed 325,515 WCLN surveillance results from tests performed from January 1, 2018 to June 30, 2024. The FilmArray GI platform was used for 42% of diagnostic tests performed by clinical laboratories during the analysis period (Table S1). Yearly percent positivity for *Campylobacter*, *Salmonella*, and STEC tested by the FilmArray GI platform was higher compared to percent positivity by other CIDTs for every year analyzed. *Campylobacter* had the highest percent positivity increase among FilmArray GI-tested specimens (average 1.56-fold increase), compared to the percent positivity increases observed for *Salmonella* and STEC (0.57- and 0.63-fold increase, respectively) (Fig. 1; Fig. S1). Quarterly analysis showed an increase in percent positivity from July to September for each year analyzed, supporting seasonal trends for *Campylobacter*, *Salmonella,* and STEC diagnosis. *Campylobacter* FilmArray GI had the highest percent positivity in quarterly analysis (Fig. S1).

### Lower *Campylobacter* culture confirmations from FilmArray GI-positive specimens

**Fig 2:**
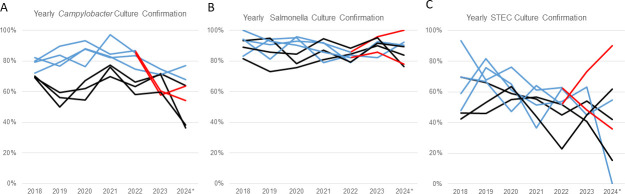
Comparison of culture confirmation of FilmArray GI versus other CIDTs. Yearly culture confirmations for *Campylobacter*, *Salmonella*, and STEC at WSLH from January 2018 to June 2024. (**A–C**) Yearly culture confirmation from high-volume submitting laboratories that use either FilmArray GI (black) or other CIDTs (blue) for organism diagnostic tests. Two laboratories switched to the FilmArrayR GI platform, with one in 2023 (blue to red transitions). *Analysis done up to June 30, 2024.

The higher percent positivity of results from the FilmArray GI compared to other CIDTs could be due to the FilmArray GI’s higher sensitivity and/or false-positive results (see Table S2 for the limit of detection for each platform). To investigate this further, we analyzed culture confirmation rates from high-volume submitting laboratories. Clinical laboratories submitted 4,460 specimens for *Campylobacter* culture from January 2018 to June 2024. Percent culture confirmations of positive CIDT *Campylobacter* tests among laboratories using FilmArray GI were significantly lower compared to laboratories using other CIDTs (average 62% vs. 78%, respectively; *P* < 0.05) (Fig. 2A; Fig. S2A). No significant differences were observed in *Salmonella* and STEC percent culture confirmations (*P* > 0.05) (Fig. 2A; Fig. S2A).

**Fig 3:**
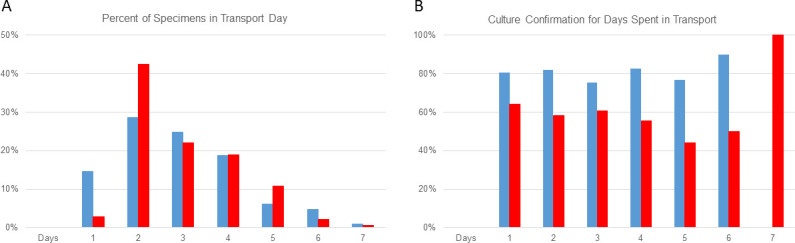
Impact of days in transport of *Campylobacter* specimens on culture confirmation. Days spent in transport is the time between the date collected by the clinical laboratory and the day received at WSLH from January 2023 to December 2023. (**A**) Percent of positive FilmArray GI specimens that spent 1–7 days in transport to WSLH (red). Percent of positive specimens by other CIDTs that spent 1–7 days in transport to WSLH (blue). (**B**) Percent culture confirmation for positive FilmArray GI specimens (red) compared to other CIDTs (blue) sent to WSLH. Logistic regression analysis *P* < 0.05.

To understand if specimen transport from clinical laboratories to WSLH affected percent culture confirmations, we analyzed the number of days *Campylobacter* stool specimens spent in transport in 2023. Time in transport was analyzed for 694 specimens. All 694 specimens spent 1–7 days in transport. The majority (604/694) of CIDT-positive *Campylobacter* specimens spent ≤4 days in transport (Fig. 3A). Percent culture confirmations of FilmArray GI-positive specimens were consistently lower than confirmations of positive specimens detected through other platforms, regardless of days spent in transport (*P* < 0.05) (Fig. 3B).

One clinical laboratory transitioned from another molecular CIDT platform to the FilmArray GI platform in spring 2023. This laboratory observed lower culture confirmations for their *Campylobacter* CIDT-positive specimens after transitioning tests to the FilmArray GI. There were no changes in culture protocol at WSLH during this time. The clinical laboratory observed a sharp decrease in percent culture confirmations with the switch to FilmArray GI: from 87% in 2022 to 60% in 2023 and 54% in 2024 (Fig. 2A). We collaborated with this laboratory and another large clinical laboratory using the FilmArray GI to further investigate this decrease in percent culture confirmations.

### Atypical melt curves for *Campylobacter* specimens with culture-negative results

**Fig 4:**
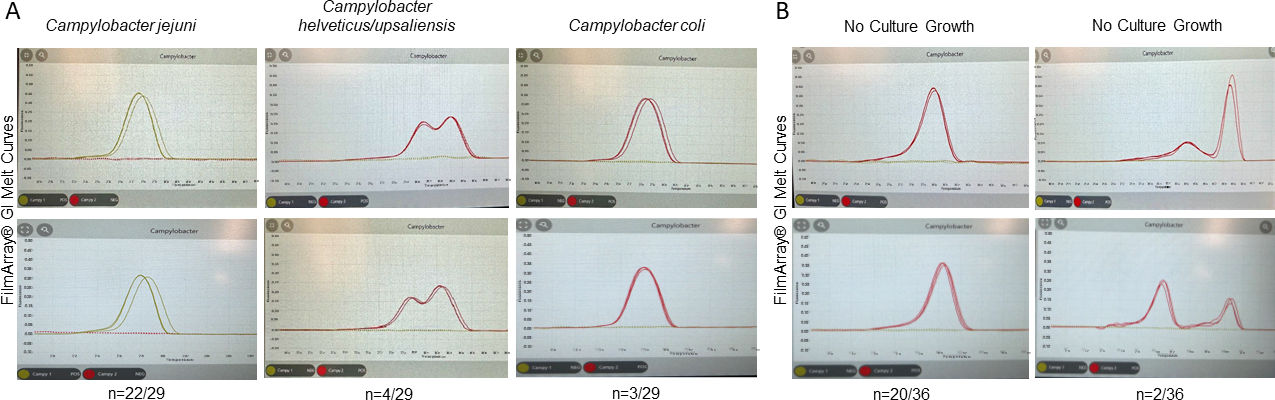
Representative FilmArray GI melt curves from two different clinical laboratories. Top: Lab 1. Bottom: Lab 2 (except *C. helviticus*/*upsaliensis* screenshots). Both are from Lab 1, as no cultures positive for those species were identified for Lab 2 during the study period. (**A**) Positive FilmArray GI melt curve for specimens with culture-confirmed *Campylobacter*. (**B**) Positive FilmArray GI melt curve for specimens with no *Campylobacter* culture growth has Campy 2 target amplification. 14/36 specimens with no culture growth had melt curves consistent with *C. jejuni*, *C. helveticus/upsaliensis*, or *C. coli* culture confirmed melt curves.

Two clinical laboratories in Wisconsin shared melt curve data from specimens that were FilmArray GI-positive. We analyzed melt curves associated with 29 cultures that grew bacterial pathogens and 36 cultures that did not grow bacterial pathogens, and results were similar from both laboratories (Fig. 4). Among 29 curves from cultures that grew bacterial pathogens, 22 were *C. jejuni*, four were *C. helveticus*/*upsaliensis*, and three were *C. coli*. Melt curves for each *Campylobacter* species were distinct (Fig. 4A). Among 36 melt curves from cultures that did not grow any bacterial pathogens, 14 melt curves matched *C. jejuni* (*n* = 6), *C. helveticus*/*upsaliensis* (*n* = 6), or *C. coli* (*n* = 2). These 14 likely represent true positives that were not able to be cultured because of low concentration or lack of live organisms. However, the majority (22/36; 61%) of cultures that did not grow any bacterial pathogens had melt curves distinct from any cultures that grew bacterial pathogens (Fig. 4B). The predominant melt curve (20/36; 56%) from cultures that did not grow any bacterial pathogens was associated with the Campy 2 target of FilmArray, with one tall peak and a long tail toward lower temperature (left side of the peak). Although similar to the *C. coli* curve, this unique signature among cultures that do not grow any bacterial pathogens has a peak at 80°C–81°C, which is higher than the *C. coli* curve that has a peak at ~78°C and does not have a long tail toward lower temperature (Fig. S3). Two additional melt curves from culture that did not grow any bacterial pathogens were uniquely abnormal (Fig. 4B).

## DISCUSSION

We performed percent positivity, culture confirmation, and melt curve analyses to assess the performance of the FilmArray GI platform for *Campylobacter* diagnosis in Wisconsin. *Campylobacter* diagnosis by the FilmArray GI increased 1.56-fold across the WCLN from 2018 to 2024 compared to other CIDTs. Some of the increase in percent positivity might be attributed to higher sensitivity in *Campylobacter* diagnosis. However, the marked decrease in percent culture confirmations from FilmArray GI *Campylobacter-*positive specimens suggests that the increase is associated with, at least in part, false-positive results. Moreover, a decrease in culture confirmation when a clinical laboratory transitioned to the FilmArray GI in 2023 further supports a platform-dependent cause, as no other protocol changes occurred. Lastly, the atypical BIOFIRE FilmArray Torch system melt curves for *Campylobacter-*positive specimens with culture that did not grow any bacterial pathogens suggest nonspecific amplification with the Campy 2 target. Taken together, the increase in percent positivity, the decrease in percent culture confirmation, and the unique signature in cultures that did not grow any bacterial pathogens suggest that aberrant melt curves are associated with false-positive *Campylobacter* results on FilmArray GI.

Although distinct, the *C. coli* melt curve had similar characteristics to the predominant culture-negative melt curve. The similarities in the Campy 2 target amplification for culture-confirmed *C. coli* and culture-negative melt curves create a challenge in validating results through melt curve analysis before reporting. These findings emphasize the need for improvement in testing platform specificity and highlight the importance of *Campylobacter* culture to aid in accurate diagnosis.

This study has several limitations, including a limited melt curve sample size (*n* = 65), especially for *C. coli,* which has a low prevalence in Wisconsin. Although *C. jejuni* and *C. coli* are all detected by the FilmArray GI and other CIDTs in this study, it is possible that the FilmArray GI also detects *C. upsaliensis*, a species that we are not always able to culture successfully. Our melt curve analysis, however, suggests that the specimens that are culture-negative at WSLH are not *C. upsaliensis*, as they have atypical melt curves. Alternatively, results may highlight differences in the limit of detection (LOD) for *Campylobacter* species by the FilmArray GI compared to other platforms. However, the average LOD across all other platforms is 4 × 10^4^ cfu/mL, similar to that of the FilmArray GI (ranges for all platforms in Table S2). We did not perform other molecular confirmation on clinical laboratory specimens that were CIDT-positive, because culture is the standard for *Campylobacter* confirmation for public health surveillance. Lastly, the laboratory-based surveillance data submitted by our clinical partners is done manually every week by those who opt in to participate and may be subject to errors or missing data, such as total specimens tested, total positive numbers, no platform entry (not a required entry), and/or missed week. To minimize errors due to incorrect reporting, only inputs with all required fields for analysis were analyzed.

CIDTs are important tools in enhancing diagnostic efforts, offering operational advantages. The FilmArray GI is one such tool that offers fast turnaround time and ease of use. However, false-positive diagnoses may have implications in patient care, such as potential for overtreatment, unnecessary antibiotic use, and strain on resources. In addition, false-positive results can impact national and state-level pathogen monitoring, making it crucial to identify diagnostic issues in order to maintain accurate surveillance. Here, we report how laboratory-based surveillance can support efforts in identifying and reporting potential issues with commercially available diagnostic tests.

## SUPPLEMENTAL MATERIAL

Figure S1 jcm.00028-25-s0001.TIF Quarterly percent positivity graphs.

Figure S2 jcm.00028-25-s0002.TIF Average quarterly percent culture confirmations graphs.

Figure S3 jcm.00028-25-s0003.TIF No culture confirmed and *C. coli* culture confirmed melt curve comparison.

Table S1 jcm.00028-25-s0004.TIF WCLN data analysis summary.

Table S2 jcm.00028-25-s0005.TIF Molecular platform LODs.

ASM does not own the copyrights to Supplemental Material that may be linked to, or accessed through, an article. The authors have granted ASM a non-exclusive, world-wide license to publish the Supplemental Material files. Please contact the corresponding author directly for reuse.
