# Supplementary figures and images for: Percentage of culture confirmation and melting curve analysis reveals false-positive Campylobacter detection in a molecular syndromic panel
Source: J Clin Microbiol. 2025 Jul 14;63(8):e00028-25. doi: 10.1128/jcm.00028-25 (PMC12345244; doi:10.1128/jcm.00028-25)

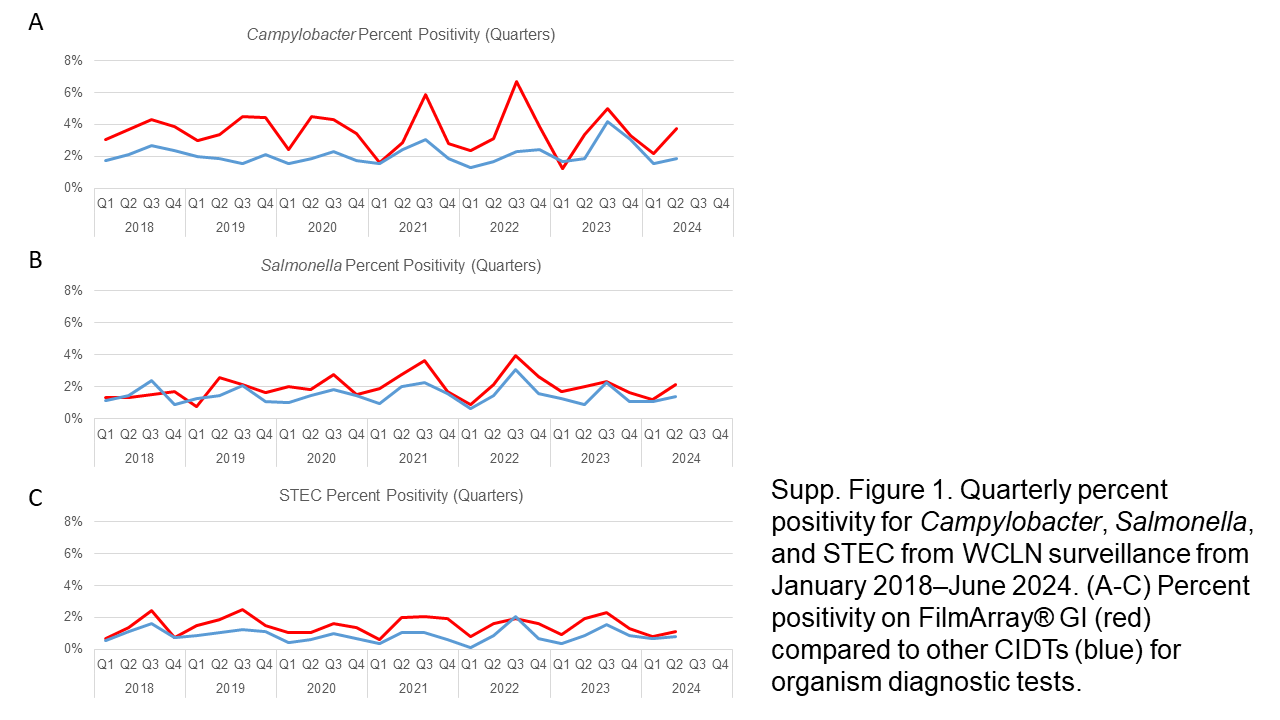

Supplement: Figure S1 — Quarterly percent positivity graphs. [file jcm.00028-25-s0001.tif]

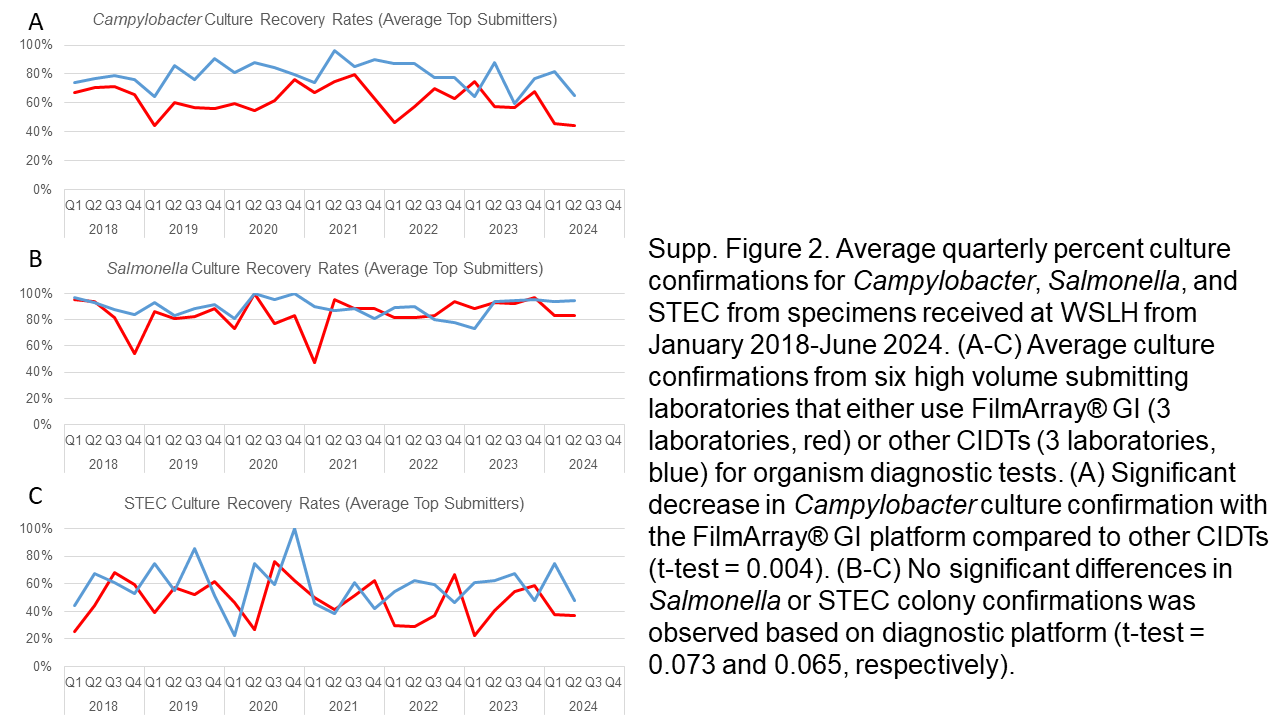

Supplement: Figure S2 — Average quarterly percent culture confirmations graphs. [file jcm.00028-25-s0002.tif]

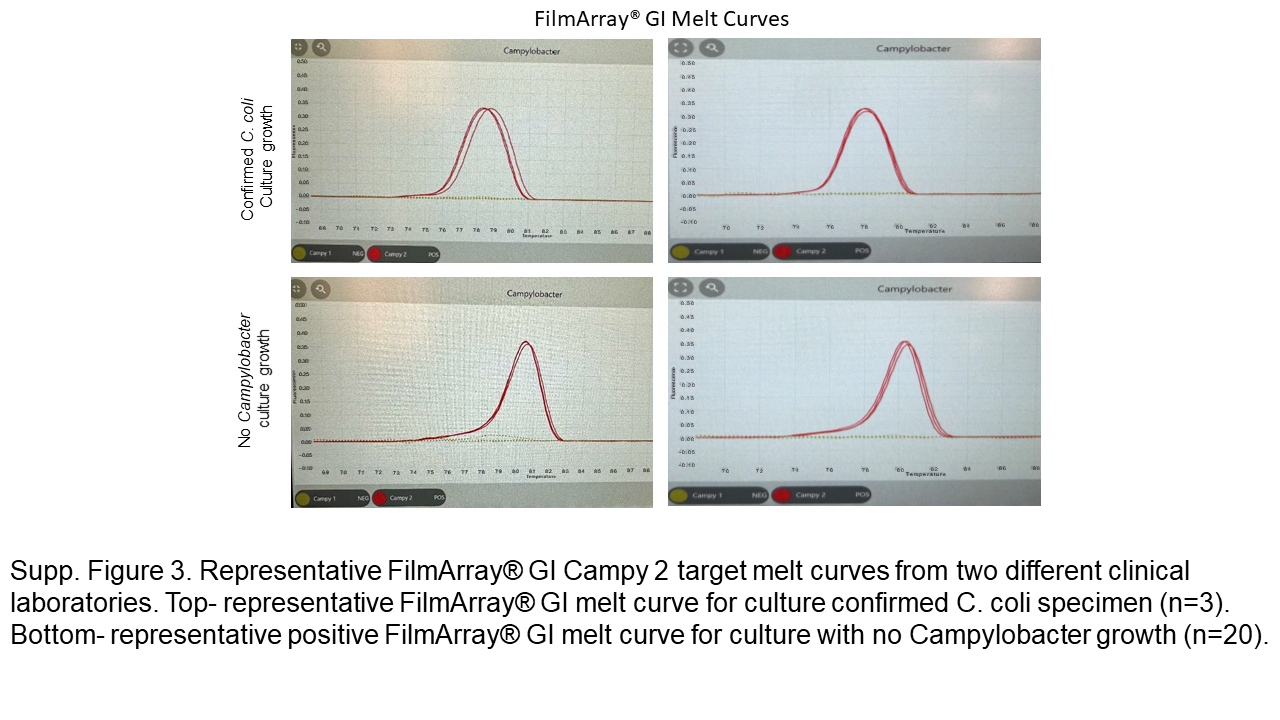

Supplement: Figure S3 — No culture confirmed and C. coli culture confirmed melt curve comparison. [file jcm.00028-25-s0003.tif]

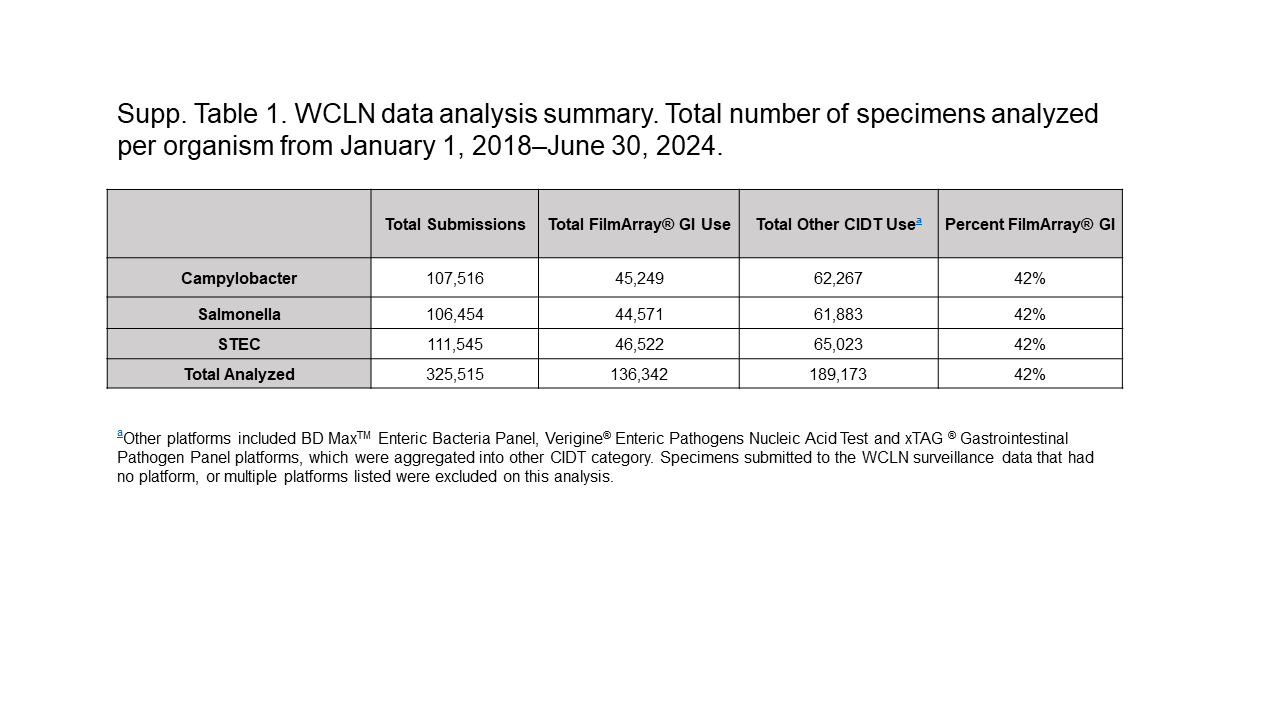

Supplement: Table S1 — WCLN data analysis summary. [file jcm.00028-25-s0004.tif]

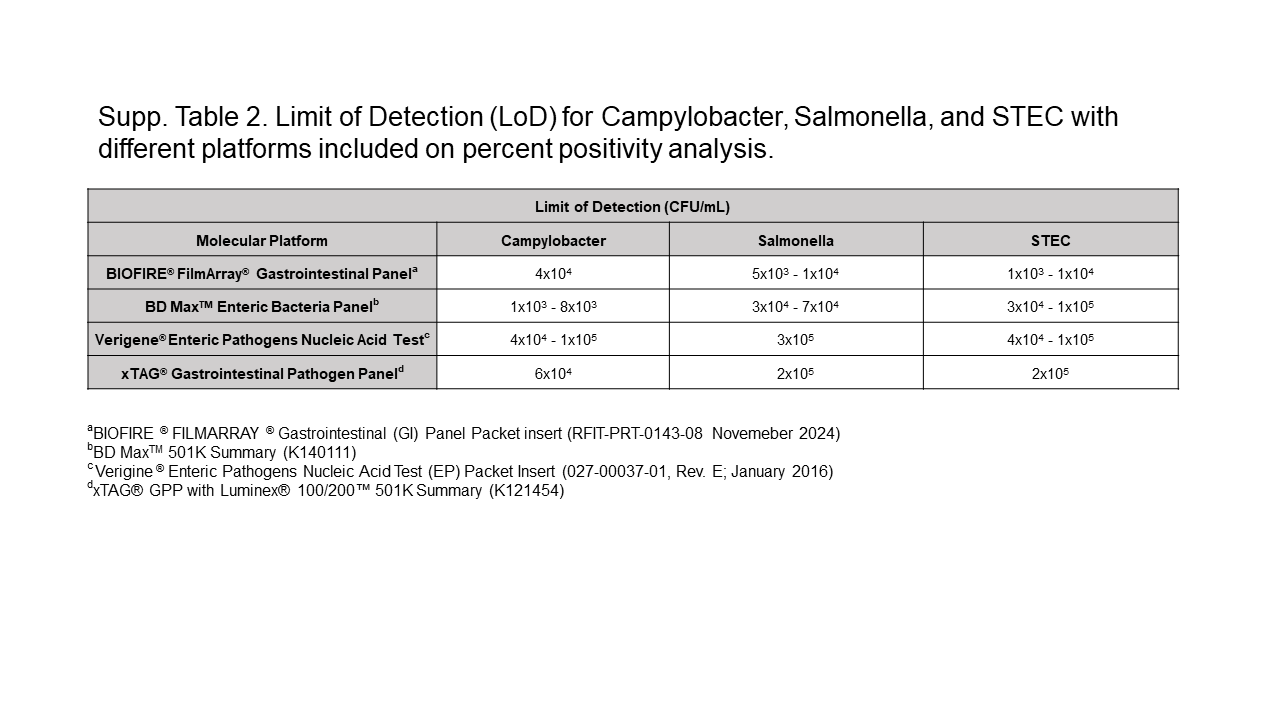

Supplement: Table S2 — Molecular platform LODs. [file jcm.00028-25-s0005.tif]
